# Connecting the dots: the role of fatigue in female infertility

**DOI:** 10.1186/s12958-024-01235-5

**Published:** 2024-06-07

**Authors:** Wenzhu Li, Xiaoyan Huang, Yiqiu Wei, Tailang Yin, Lianghui Diao

**Affiliations:** 1 Reproductive Medical Center, Renmin Hospital of Wuhan University and Hubei Clinic Research Center for Assisted Reproductive Technology and Embryonic Development, Wuhan, 430060 China; 2 Department of Rheumatology, The University of Hong Kong- Shenzhen Hospital, Shenzhen, 518053 China; 3 Shenzhen Key Laboratory of Reproductive Immunology for Peri-implantation, Shenzhen Zhongshan Institute for Reproductive Medicine and Genetics, Shenzhen Zhongshan Obstetrics & Gynecology Hospital (formerly Shenzhen Zhongshan Urology Hospital), Shenzhen, 518045 China; 4 Guangdong Engineering Technology Research Center of Reproductive Immunology for Peri- implantation, Shenzhen, 518045 China

**Keywords:** Female infertility, Fatigue, Stress, HPA axis, Inflammation

## Abstract

Fatigue, an increasingly acknowledged symptom in various chronic diseases, has garnered heightened attention, during the medical era of bio-psycho-social model. Its persistence not only significantly compromises an individual’s quality of life but also correlates with chronic organ damage. Surprisingly, the intricate relationship between fatigue and female reproductive health, specifically infertility, remains largely unexplored. Our exploration into the existing body of evidence establishes a compelling link between fatigue with uterine and ovarian diseases, as well as conditions associated with infertility, such as rheumatism. This observation suggests a potentially pivotal role of fatigue in influencing overall female fertility. Furthermore, we propose a hypothetical mechanism elucidating the impact of fatigue on infertility from multiple perspectives, postulating that neuroendocrine, neurotransmitter, inflammatory immune, and mitochondrial dysfunction resulting from fatigue and its co-factors may further contribute to endocrine disorders, menstrual irregularities, and sexual dysfunction, ultimately leading to infertility. In addition to providing this comprehensive theoretical framework, we summarize anti-fatigue strategies and accentuate current knowledge gaps. By doing so, our aim is to offer novel insights, stimulate further research, and advance our understanding of the crucial interplay between fatigue and female reproductive health.

## Introduction

Infertility is a condition characterized by the inability of a woman to conceive after a year of regular, unprotected intercourse. It poses a significant global health concern, affecting approximately 15% of couples worldwide, with female factors alone accounting for at least 35% [1–3]. Beyond the physiological challenges, long-term infertility can lead to emotional distress, strained relationships, and societal stigma, impacting the overall well-being of couples aspiring to have children [4].

Fatigue, commonly described as extreme tiredness, diminished energy, and reduced physical and mental capacity, is a prevalent symptom in various chronic conditions [5]. While there is no consensus definition, and it overlaps with factors such as chronic pain, physical exertion, sleep disorders, and psychological stress [6, 7]. Under the bio-psycho-social medical model, the concept of “whole-person care” has been increasingly accepted and applied, and patient-reported outcomes (PROs) have received more attention in clinical research, with fatigue being an important indicator of disease [8–10]. This phenomenon has also appeared in the field of gynecology, thus, we began to focus on the impact of fatigue on infertility.

There is growing recognition of the potential link between fatigue and female infertility, but scientific evidence remains limited. Fatigue could impact fertility through complex mechanisms involving the neuroendocrine system [11], inflammatory-immune response [12], energy metabolism, and oxidative stress [13]. Furthermore, the development of fatigue is associated with changes in neurotransmitter metabolism and neuronal plasticity [14]. These alterations, in turn, may affect crucial reproductive processes like ovulation, implantation, and embryonic development. What’s more, managing chronic fatigue can exact a significant emotional toll, potentially leading to stress, anxiety, and depression, which may further impact fertility through hormonal imbalances [15], disrupted menstrual cycles [16], and decreased sexual desire [17].

**Fig. 1:**
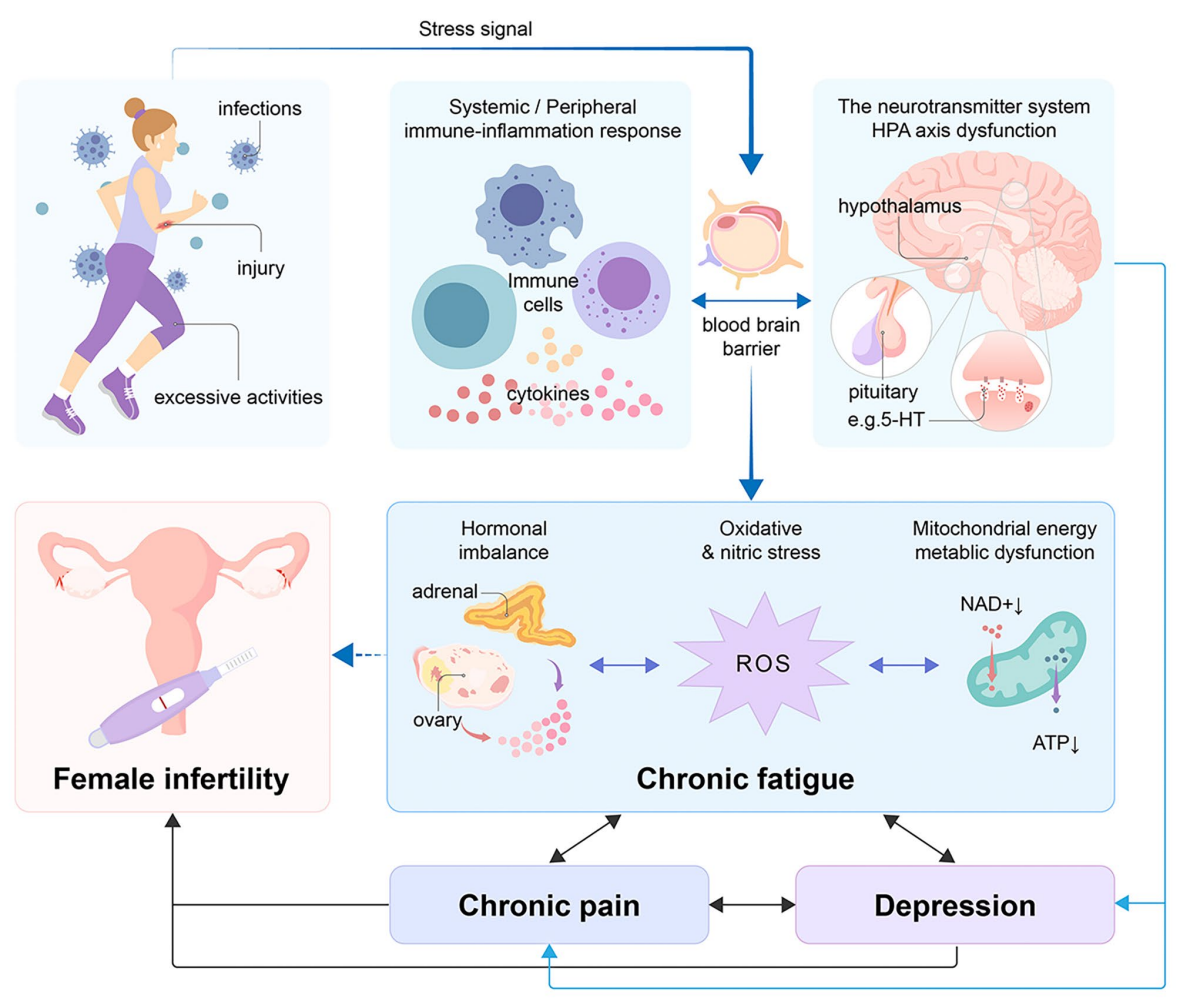
Potential mechanisms by which fatigue influences female infertility. The relationship between fatigue and female infertility is under-recognized, and it has complex crosstalk with co-factors (for example, pain and depression) through the activation of in vivo stress-responsive systems (HPA-axis, neurotransmitter system) and inflammation stimulated by different stress signals, and then contributing to hormonal dysregulation, oxidative stress, and mitochondrial metabolic dysfunction, which may be an underlying mechanism that affects female infertility. The solid lines represent reported studies, and the dotted lines represent studies that need to be developed. Double arrows represent interconnections. HPA, hypothalamic-pituitary-adrenal; 5-HT, 5-hydroxytryptamine; ROS, reactive oxygen species

In this review, we aim to provide a comprehensive overview of the link between fatigue and female infertility, delving into the potential mechanisms through which fatigue impacts reproductive health (Fig. 1). By connecting the dots between fatigue and factors closely tied to infertility, we endeavor to unveil the complex interactions and indirect effects of fatigue on female fertility. Our objective is to offer novel insights, stimulate further research, and propel the advancement of our comprehension in this pivotal realm of female reproductive health for the benefit of graduate students in medicine and practicing physicians.

### Epidemiology of fatigue

Fatigue is a multifaceted symptom experienced by both healthy and unhealthy individuals, lacking a clear-cut definition. Self-reported scales are the primary measurement tools [18]. Current research suggests a key distinguishing factor between healthy and disease-related fatigue is the inability to alleviate fatigue with rest [19]. Fatigue often occurs as a comorbidity alongside various psychophysical factors, such as anxiety, depression, and pain, as demonstrated by multivariate analyses [20–26]. Therefore, exploring the influence of these co-factors on infertility is vital.

Though not life-threatening, fatigue severely affects the quality of life (QoL) of infertile women [27]. Despite regional variations in reported incidence [28–32], a recent meta-analysis estimated the global prevalence of general fatigue at 20.4% in adults and 11.7% in minors [33]. Studies from 2011 highlighted that 7–45% of the U.S. population experiences persistent fatigue [34]. A cross-sectional study involving 2063 individuals showed that over 50% of patients were troubled by fatigue in Saudi Arabia [35]. Importantly, women appear to be more susceptible to fatigue than men, with a pooled odds ratio [33]. Further, studies support that female sex may be an independent risk factor for persistent fatigue [28, 36].

Fatigue can manifest throughout a woman’s reproductive life cycle, impacting sexual maturity, pregnancy, post-partum, and perimenopause. Postpartum fatigue is well-documented [37, 38], but there’s a paucity of research on fatigue in infertile women. However, fatigue in women of reproductive age, especially in infertile women, has only begun to be mentioned in recent years. Approximately 25–60% of infertile patients are reported to be disturbed by psychological factors, including anxiety and depression [39], but the prevalence of fatigue in infertility is understudied. An observational study showed that 52 out of 140 infertile women had self-reported fatigue, and the severity of fatigue negatively affected their QoL [40]. Similarly, a study involving 149 infertile patients showed that fatigue was the most influential factor in the QoL of infertile women [41]. Taken together, more research is critically needed to address this gap.

### Current state-of-the-art of fatigue in female infertility

Research on how fatigue directly impacts female infertility remains limited. However, fatigue is discussed for several chronic conditions affecting the uterus and ovaries (Table 1). Additionally, emerging studies suggest that autoimmune diseases, characterized prominently by fatigue, may adversely affect female fertility. Overall, research on fatigue associated with infertility faces multiple challenges and barriers.

**Table 1:** Characteristics of the Typical Studies Included in the Review

Chronic Conditions	Author Information	Study Type/ Sample Size	the Role of Fatigue	Fatigue Measurement
Endometriosis	Facchin et al. 2021 [45]	Matched pair case-control study (EMT, *N* = 123; Control, *N* = 123)	Women with painful EMT reported significantly greater fatigue	5-point Likert scale
	Álvarez-Salvago et al. 2020 [49]	Matched pair case-control study (EMT, *N* = 25; Control, *N* = 25)	Women with higher endometriosis-related fatigue had a lower QoL.	The Spanish version of the Piper Fatigue Scale
	Mundo-López et al. 2020 [48]	Cross-sectional study (EMT, *N* = 230)	One-third and one-half of the recruited patients with EMT showed moderate to severe fatigue, which is closely related to higher anxiety and depression, poorer sleep quality, lower sexual function, and so on by regression analysis.	Piper Fatigue Scale
	Ramin-Wright et al. 2018 [43]	Multi-center matched case-control study (EMT, *N* = 560; Control, *N* = 560)	More women diagnosed with EMT experienced frequent fatigue than control women. Fatigue in EMT was associated with insomnia, depression, pain, and occupational stress by regression analysis.	The self-administered questionnaire developed by endometriosis and psychosomatic specialists from the universities of Zurich and Berlin.
Polycystic Ovary Syndrome	Boivin et al. 2020 [52]	NA 120(PCOS, *N* = 11; PCOS screen-positive, *N* = 25; PCOS screen-negative, *N* = 74)	The PCOS-confirmed women scored more poorly than control groups on physical, emotional, social, and spiritual well-being indexs, like fatigue and depression.	Fatigue Symptom Inventory
	Abdollahi et al. 2019 [51]	Randomized controlled clinical trial (PCOS with CBT, *N* = 37; PCOS, *N* = 37)	Cognitive-behavioral therapy (CBT) significantly reduced psychological fatigue in PCOS patients.	The Fatigue Impact Scale
Premature Ovarian Insufficiency	Benetti-Pinto et al. 2019 [57]	Cross-sectional study (POI with HT therapy, *N* = 61; Control, *N* = 62)	The POI group had a higher fatigue index than controls.	Chalder Fatigue Scale
	Huang et al. 2021 [56]	Cross-sectional study (POI, *N* = 293; Control, *N* = 471)	Fatigue is one of the 13 items of menopause. 168 of 293 women with POI suffered from fatigue (57.3%).	The modified Kupperman Menopausal Index
	Ates et al. 2022 [58]	Matched pair study (POI, *N* = 62; Control, *N* = 62)	Fatigue did not differ significantly between the groups	Fatigue Severity Scale
Premature Ovarian Failure	Stege et al. 2008 [59]	Case-control study (POF, *N* = 81; Control, *N* = 68)	Fatigue did not differ significantly between the groups	The Shortened Fatigue Questionnaire

### Fatigue in Uterine and Ovarian diseases

#### Endometriosis

Endometriosis (EMT), defined as the presence of endometriotic lesions outside the uterus, is a greatly disturbing gynecologic disease that affects about 10% of women of reproductive age [42]. As early as 2018, a multicenter cross-sectional study sponsored by Wright and his colleagues found fatigue as a frequent symptom of EMT and was closely associated with insomnia, depression, pain, and occupational stress [43]. Subsequently, many observational studies have documented similar results [44]. Several evidence have indicated that the severity of EMT is associated with increased fatigue. A matched pair case-control study revealed that women with painful endometriosis reported significantly greater fatigue than those without significant pain symptoms and controls [45]. Ashrafi et al. found fatigue to be a predictor of risk for EMT through multiple logistic regression [46]. EMT-related fatigue seriously undermines women’s sense of life well-being and even their relationships with partners [47]. A scale-based observational study in Spain emphasized that moderate to severe fatigue contributed to psychological impairment, such as higher anxiety and depression, poorer sleep quality, and lower sexual function [48]. The above studies showed that fatigue is a common, secondary symptom of EMT, and is related to other somatic and psychological impairments, ultimately significantly decreasing patients’ QoL and sexual function [49].

#### Polycystic ovary syndrome

Polycystic ovary syndrome (PCOS) is the most common chronic endocrine disease characterized by menstrual dysfunction and ovulation disorders affecting 5–13% of women in the general population [50]. Studies have found that PCOS women often suffer from increased anxiety and depression, causing psychological fatigue and further impairing QoL [51, 52]. It is noted that patients with lower QoL parameters showed more worse marital sexual functioning [53]. A randomized clinical trial found that a psychological treatment named cognitive behavioral therapy (CBT) can significantly reduce the severity of fatigue in PCOS patients compared to the control group [51]. On the whole, fatigue almost goes unnoticed in patients with PCOS [54].

Premature Ovarian Insufficiency.

Premature ovarian insufficiency (POI) develops the breakdown of ovarian function and menopause before the age of 40, affects about 1% of women, and is one of the main causes of female infertility [55]. A cross-sectional study showed fatigue was a highly prevalent symptom closely associated with menopause in the recruited Chinese women with POI [56]. A few cross-sectional studies supported that the POI group had a higher fatigue index than controls [56, 57]. In another study, POI patients were more likely to suffer from sleep disturbance and depression, but not fatigue compared to age-matched healthy individuals [58]. Similar results were also obtained in an earlier study on premature ovarian failure [59]. Thus, the available evidence on whether patients with POI are at higher risk for fatigue is heterogeneous and needs to be studied extensively.

### Fatigue in Autoimmune diseases affecting fertility

Autoimmune diseases are a large group of chronic disorders mediated by immune responses to self-antigens, such as rheumatoid arthritis (RA) [19], antiphospholipid syndrome [60], systemic lupus erythematosus [61], multiple sclerosis (MS) [62], autoimmune hypothyroidism [63]. A range of mental and somatic symptoms are present in these disorders, with fatigue being one of the most common and challenging symptoms to manage [64]. Fatigue is often indicative of disease activity in these disorders [19], while disease activity raises the risk of adverse pregnancy outcomes, particularly miscarriage [65–67]. MS patients with fatigue had significantly higher levels of C-reactive protein (CRP) during pregnancy than those without fatigue, suggesting that higher CRP, an indicator of systemic inflammation, may be somewhat predictive of pregnancy-related comorbidities [68]. A small single-center retrospective study revealed that more patients with MS underwent elective cesarean deliveries due to fatigue [69]. Additionally, the state of chronic fatigue directly impairs sexual function of women, which contributes significantly to the probability of fertility [17, 70], For instance, some studies showed sexual function in women with RA may be affected by pain and fatigue, potentially impacting fertility [71, 72]. Despite the shared occurrence of fatigue and female infertility in autoimmune diseases, high-quality research investigating the impact of fatigue on infertility in these conditions is needed.

### Challenges of Fatigue Research in female infertility

The underrepresentation of fatigue research within female infertility remains a significant obstacle. This can be attributed to several factors:

#### Subjective and difficult measurement

The qualitative assessment of fatigue is relatively difficult and often subjective, and relies on the use of subjective self-report questionnaires [18, 73]. Moreover, experts in different fields may develop professional-specific questionnaires [74, 75]. The lack of standardized fatigue measurement across studies, including variations in the specific scales, recall periods, and wording, makes it challenging to compare results and draw definitive conclusions.

#### Focus on cross-sectional designs

Most of the studies on fatigue and infertility utilizes cross-sectional designs [76–78], which typically establishes causality through multiple regression analysis. The cross-sectional designs limit the ability to establish clear cause-and-effect relationships.

#### Potential confounding factors

Difficulties in fatigue research also arise from potential confounders of fatigue, such as emotional distress and pain [19, 79], suggesting that fatigue is not a single symptom of infertility, but rather is accompanied by a number of psychological and physical symptoms [40]. It is paramount to consider these interconnected factors to fully understand the complex relationship between fatigue and infertility.

### Putative mechanisms of fatigue on female infertility

Longstanding fatigue serves as a catalyst for pathophysiologic changes in numerous systems and organs [11], posing detrimental effects on health [80], which may become the key force driving infertility. The pathogenesis of fatigue, involves the central nervous system (CNS) and autonomic nervous system (ANS), immune and inflammation, mitochondrial oxidative stress [81–83]. Simultaneously, the mechanisms of infertility are closely related to neuro-endocrine dysregulation, inflammatory and immune imbalances, and oxidative stress activation [62, 84]. It is clear that there are shared mechanisms for fatigue and infertility, which is worth exploring. Consequently, the forthcoming section presents evidence in support of a hypothesized mechanism illustrating overlap between fatigue and infertility.

### Neuroendocrine disruption and Hormonal Imbalance

The CNS and endocrine system collaborate to maintain daily activities and keep up energy. Structural and functional dysfunctions in different regions of the brain may disrupt motor cortical excitability, hormone secretion, and the signal to the working muscles, which in turn cause fatigue [85, 86]. In particular, abnormalities in the hypothalamic-pituitary-adrenal (HPA) axis have been identified as a cause of fatigue. Notably, adrenal hormone dysregulation, such as hypocortisolemia, is frequently found in fatigued individuals, representing one facet of the imbalance of the HPA axis [87]. For instance, one study found MS patients with fatigue had significantly higher pituitary heights and widths, along with increased secretion of adrenocorticotropic hormone compared to patients without fatigue. These findings suggest CNS and endocrine systems are involved in the pathogenesis of fatigue through sophisticated regulation of brain structure and function [75].

More importantly, it is closely tied to maintaining reproductive function. First of all, gonadotrophin-releasing hormone (GnRH) from the hypothalamus, along with luteinizing hormone (LH) and follicle-stimulating hormone (FSH) from the pituitary gland, regulates follicular development and the ovarian cycle [88]. What’s more, the HPA axis has a direct inhibitory action on the hypothalamic-pituitary-ovarian axis in certain ways [15], impacting the cyclical patterns in central and ovarian hormones, including FSH, LH, E2, and P4. Besides, one early study found that uterine cortisol deficiency may mediate elevated levels of NK cells, which further impaired decidualization [89]. Dehydroepiandrosterone (DHEA), a kind of adrenal hormone, is linked to fatigue and turns out to be an important contributor to reproductive function [90]. DHEA deficiency can severely impact ovarian hormone synthesis and ovarian reserve, potentially leading to premature ovarian insufficiency (POI) [91]. Supplementation with DHEA has demonstrated positive effects on pregnancy rates, emphasizing its role in maintaining fertility [92–95]. Beyond the HPA axis, hormonal changes associated with fatigue extend to estrogen, progesterone and thyroid hormone [96–99]. Moreover, untreated maternal hypothyroidism is associated with adverse pregnancy outcomes such as miscarriage, preeclampsia, and many neonatal disorders [100].

In fact, although fatigue is a rather ill-defined physical manifestation and the relationship with female infertility has not been directly studied, evidence of neuroendocrine disruption and hormonal imbalance found in fatigue-related disorders supports their relevance to the maintenance of female fertility.

### Stress and nervous system response

Perhaps, it is reasonable to suggest that fatigue and its co-factors can contribute to infertility via shared mechanistic pathways. Organisms are required to respond and adapt adequately to ubiquitous stress in an ever-changing environment, such as viral infections, trauma, and adverse life events. In the first place, the onset of fatigue and its co-factors is regulated by the in-vivo stress response system [101, 102]. When subjected to internal and external stress, the biological stress response system immediately makes corresponding alterations, including the HPA axis and the ANS system, as well as the neuroimmune system [14, 103–105]. Sustained stress may lead to maladaptive responses manifested as dysregulation of the above systems [106], which is specifically manifested as abnormal levels of serum catecholamines and glucocorticoids and persistent low-grade inflammation based on findings such as microglial activation in the brain and increased pro-inflammatory cytokines in the periphery [107]. Moreover, stress and stress hormones inhibit the release of GnRH, and glucocorticoids suppress LH secretion [108]. These abnormal alterations may further disrupt ovarian steroidogenesis and exacerbate inflammatory storms, ultimately being detrimental to female fertility.

Anhedonia attributed to deficits in reward processing is a shared feature in many neuropsychiatric symptoms, including fatigue, depression, and chronic pain [62]. The processing ability of brain reward circuits is disrupted by inappropriate levels of monoaminergic neurotransmission, mainly including serotonin (5-HT), norepinephrine, and dopamine with their degenerate mesocorticolimbic pathways from the midbrain to the basal ganglia, the limbic system, and the prefrontal cortex [62, 109, 110]. The lack of neurotransmitters, including 5-HT, can cause sexual dysfunction, thereby reducing fertility opportunities [15, 111].

Overall, hormonal imbalances and incorrect release of neurotransmitters caused by the nervous response system could result in infertility. Additionally, fatigue could reduce sexual desire and cause sexual dysfunction, ultimately declining the chance of pregnancy [48]. The above evidence suggests that fatigue mediate neurologic dysregulation that can aggravate infertility.

### Low-grade inflammatory activation

Of great interest, fatigue is common in chronic autoimmune, inflammatory diseases [112, 113]. A recent cross-sectional study found elevating fatigue severity is closely linked to stronger signs of monocyte activation, including increased inflammatory gene expression in monocytes, higher CD8^+^ T-lymphocyte counts, and increased serum pro-inflammatory cytokines [114]. There is a strong correlation between fatigue severity and production of pro-inflammatory cytokines [19]. Women with fatigue often show multiple immune dysfunctions rendering them susceptible to upper respiratory tract infection, chronic lymphadenopathy, and high body temperature [115]. These dysregulations involve cell-mediated immunity, including impairment of the function of NK cells, hypo-reactivity of T cells to the antigen, the activation of monocyte macrophages, and the persistence of autoreactive cells [116]. Concurrently, they manifest as changes in many inflammation-related markers, such as increased CRP levels [68], elevation in pro-inflammatory factors like interleukin-1β, interleukin-6, interleukin-12, interleukin-2, tumor necrosis factor-alpha and interferon-gamma, increased expression of nuclear factor kappa-B, as well as decreased levels of anti-inflammatory factors like interleukin-8, interleukin-13, interleukin-15, and interleukin-23 [117]. The changes in inflammation-related profile support that patients experiencing fatigue are in a low-grade inflammatory state. In a word, inflammatory activation is undoubtedly a key step in the onset of fatigue [118].

It was reported that the increased infertility rate in RA with fatigue symptoms is due to an imbalance of inflammatory factors [72]. Indeed, the immune system serves as a crucial bridge connecting various systems within the organism, with cytokine and immune cells distributed in both in center and periphery systems. The homeostasis of immunity and inflammation is necessary to maintain a successful pregnancy. The imbalance of inflammatory factors and immune cell profiles is a notable phenomenon in patients with adverse pregnancy history [119, 120]. Such abnormalities could significantly impact pregnancy outcomes by damaging ovarian function [121], inhibiting endometrial receptivity [122], hindering trophoblast development, and interfering with immune tolerance at the maternal-fetal interface [65].

In short, inflammation is one of the most common mechanisms underlying fatigue and is also a known contributor to infertility. Fatigue may indicate active inflammation that needs to be managed in the reproductive field.

### Mitochondrial dysfunction and Cellular stress

Fatigue is defined as “a lack of energy”, indicating a close relationship with energy metabolism [13], which is manifested as impacted adenosine triphosphate production [123]. Mitochondria are core organelles that have existed in most cells since biological evolution. They provide continuous energy for biological survival and function, produce adenosine triphosphate and lactic acid, and maintain the production level of reactive oxygen species (ROS) and reactive nitrogen species (RNS) and the balance of calcium and iron in cells, removing peroxides in time. Dysfunctional mitochondria can generate and release mitochondrial components, including cardiolipin, mitochondrial DNA, and mitochondrial formylated peptides. These components, once released, can act as damage-associated molecular patterns, triggering an inflammatory response through the activation of pattern recognition receptors [124]. In addition, mitochondria play a rate-limiting role in controlling steroidogenesis, and mitochondrial impairment in neurons further affects the synthesis of neuroactive steroid hormones in the brain [125]. And the collapse of mitochondria at neuronal synapse impedes the release of neurotransmitters [126, 127]. In summary, loss of neuroendocrine-immune homeostasis due to mitochondrial disruption may partially explain infertility.

Furthermore, mitochondria function as the primary origin of ROS/RNS as byproducts of nutrient metabolism. This occurrence is concomitant with various indicators of oxidative stress, including diminished activity of antioxidant enzymes such as superoxide dismutase and plasma-like glucose peroxidase, as well as reduced levels of zinc. Conversely, there is an elevation in the activity of pro-oxidative enzymes like myeloperoxidase, along with increased levels of nitro-tyrosine and heightened nitric oxide production [118]. The heightened presence of ROS/RNS assumes a pivotal role in the manifestation of fatigue. A study demonstrated that the accumulation of ROS/RNS during exercise had a detrimental impact on Na^+^/K^+^- ATPase activity, calcium conversion and sensitivity in myofibrils, and actin-myosin dynamics. These effects collectively resulted in a reduction in the generation of muscle energy, ultimately culminating in the onset of muscle fatigue [128]. Similarly, it has been proved that many experimental drugs with antioxidant properties can improve and alleviate chronic fatigue-like behaviors by changing ROS signaling pathways [129, 130]. However, excessive oxidative stress could impair the female reproductive system by destroying oocyte quality [131, 132], damaging endometrial receptivity [133], promoting trophoblast apoptosis, medicating implantation failure, and early pregnancy loss [134]. Thereby, we believe mitochondrial dysfunction and cellular stress, closely linked with fatigue, exert a profound influence on the function of the reproductive system, but need to be further studied.

Taken together, although explained from multiple perspectives, the current evidence is still insufficient to prove whether fatigue is a result or a predisposing factor for female infertility. The above hypothesized mechanisms regarding the impacts of fatigue on infertility perhaps only provide new viewpoints for researchers in the future.

### The potential benefits of fatigue management on infertility

**Fig. 2:**
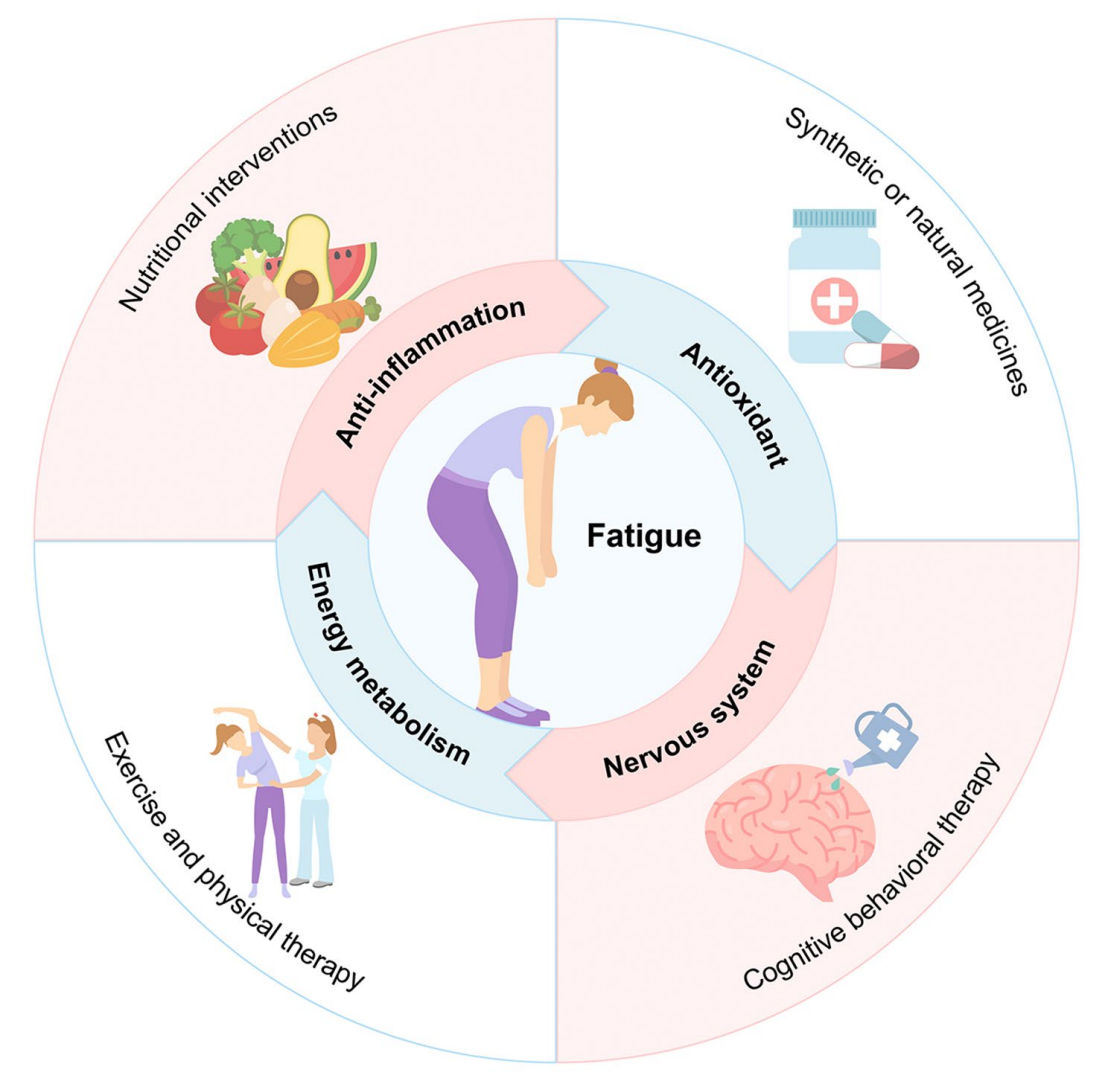
Strategies as well as mechanisms to alleviate fatigue. There are four main measures of fatigue alleviation: dietary supplements, medication, exercise and physical therapy, and psychological interventions. Relief of fatigue is achieved through anti-inflammatory, antioxidant, improved energy metabolism, and neurological modulation. The circular arrows indicate that the anti-fatigue mechanisms interact with each other and do not correspond to specific anti-fatigue measures

Given the potential connection between fatigue and infertility, it’s reasonable to consider whether fatigue management could benefit those experiencing infertility. An early intervention for fatigue is recommended, as a systematic review pointed out health risks associated with fatigue can occur earlier than hospitalization, illness, and death [135]. Current treatments for fatigue include dietary supplement/nutritional interventions, medications, exercise, physical therapy, and psychological interventions [136–140] (Fig. 2). Among them, some strategies have been reported to be beneficial for treating infertility, while others may be in favor of the health of female reproduction.

#### Anti-inflammatory and antioxidant nutrients

These nutrients, such as coenzyme Q10, L-carnitine, iron, zinc, methionine, nicotinamide adenine dinucleotide, and vitamins, help relieve fatigue [141–143]. Some studies have shown that supplementation with iron can significantly improve fatigue in women of reproductive age, potentially contributing to achieving successful pregnancy [144, 145]. Meanwhile, they belong to mitochondria-targeted nutrient therapy, which improves oxidative damage to ovarian and uterus. For instance, coenzyme Q10 is a key component of ATP production via oxidative phosphorylation, and improves mitochondrial membrane potential and superoxide levels, thereby rescuing ovarian reserve deficiency [146]. Zinc supplementation has been reported to significantly improve oocyte glutathione and mitochondrial activity, as well as reduce ROS levels, which facilitates oocyte maturation [147]. Vitamins have been shown to increase mitochondrial biosynthesis [148], and restore endocrine and metabolic homeostasis [149], which in turn improves follicular development and subsequent follicular quality in the PCOS model.

#### Medications

Some synthetic and natural drugs can combat fatigue. Glucocorticoids, relieve fatigue due to hypocortisolism and may improve ovarian function to treat infertility through regulating neuroendocrine function [150, 151]. Synbiotics, a bio-mixture of probiotics and nutrients, show potential in treating multiple symptoms of fatigue, including improving inflammation and stress responses mediated by the cytokine-HPA axis, along with mental and physical health [152], and enhance energy metabolism effectively [139].

#### Exercise and Physical Therapy

In addition to medication, exercise rehabilitation is used to reduce fatigue. A recent meta-analysis found that physical therapy was efficacious and safe in reducing fatigue in people with inflammatory rheumatic and musculoskeletal diseases [64]. It is believed that practicing yoga has a positive effect on reproductive organs and increases blood circulation [153]. Acupuncture treatment for infertile patients can regulate menstrual cycle and reduce fatigue, thus facilitating the process of ART cycles [154].

#### Psychological interventions

Furthermore, psychological interventions, particularly CBT, are applied in the management of people with fatigue [155]. The psychological vulnerability screening and additional psychological consultation for infertile women are endorsed to mitigate these mixed symptoms that are not conducive to pregnancy, such as fatigue, depression, and anxiety [156]. A quasi-experimental study found psychological interventions significantly reduced depression and fatigue of infertility women, in turn, improved their intimacy and sexual satisfaction [157]. Two randomized controlled trials from Brunei found that Nursing care reduced depression and fatigue, and improved feelings of social support and sleep quality among infertile women [158, 159]. It can be seen that psychotherapy is one of the recommended treatments for infertility patients. Herein, it is conceivable that measures to alleviate fatigue may be beneficial in treating infertility and improving reproductive outcomes.

### Current gaps and future perspectives

The direct connection between fatigue and female infertility remains weak in evidence. Considering our previous discussion, it is apparent that fatigue alone is not the singular determining factor in female infertility. Unfortunately, the current concept of fatigue is vague [160], and the diagnostic criteria are not unified [161]. In different studies, fatigue can serve as an experimental concept, a symptom, a risk, a cause, and a result [6]. Moreover, the scales for assessing fatigue are varied [74, 75] and relatively subjective. There are three models for inducing fatigue, including exhaustive exercise-induced fatigue, and chemotherapy or radiotherapy-induced fatigue [162, 163]. These heterogeneities increase the difficulty of conducting research, thus making it challenging to explore fatigue in female infertility. Unlike depression, fatigue is often a concomitant symptom and is not subjectively valued, despite its significant impact on women’s QoL and reproductive health.

While anti-fatigue interventions might benefit female infertility, current studies lack thorough mechanistic exploration. The rational use of anti-fatigue approaches and their impact on female infertility and pregnancy outcomes need to be further investigated [139, 164].

In summary, the understanding of “fatigue” is weak when it comes to reproduction. Exploring the relationships between fatigue, female infertility, and adverse pregnancy outcomes is crucial, requiring more investigation in clinical trials and basic research. In clinical trials, utilizing established fatigue assessment scales and comprehensive recording of influencing factors are advised. Rigorous statistical methods, including machine learning and multivariate logistic regression, should be employed to analyze the relationship between fatigue and female infertility or pregnancy outcomes, under the premise of eliminating confounding factors. It is important to develop strict inclusion criteria for clinical populations with fatigue symptoms, implement subgroup analysis, and explore the benefits of fatigue management on fertility outcomes. Additionally, biomarker detection in blood and other humor, such as uterine fluid, may reveal insights into fatigue and pregnancy outcomes. In basic research, the use of well-established fatigue-related animal models is essential to investigate their potential impacts on reproduction.

## Conclusion

Fatigue, a perplexing and disabling symptom, has gained more recognition in the medical era of bio-psycho-social model amid heightened individual psychological stress. It is thought to arise from endocrine imbalance and neurotransmitter changes in the central nervous system, immune-inflammation disruption, mitochondrial dysfunction, and excessive oxidative stress during viral infections or social-environmental stress. It is closely linked to both psychological and somatic factors, indirectly affecting female infertility. Prolonged fatigue can significantly diminish women’s quality of life and reproductive health, yet public awareness and understanding of this issue remain limited. Our study highlights the associations between fatigue and several common chronic conditions, and proposes hypotheses regarding the impact of fatigue on female infertility with the hope of calling attention to the issue of fatigue in infertile women in alignment with the whole-life care concept and providing insights for future study in this topic.

## Data Availability

No datasets were generated or analysed during the current study.

## Abbreviations

HPA: Axis: Hypothalamic-Pituitary-Adrenal Axis
QoL: Quality of Life
MS: Multiple Sclerosis
IVF: In Vitro Fertilization
EMT: Endometriosis
PCOS: Polycystic Ovary Syndrome
CBT: Cognitive Behavioral Therapy
POI: Premature Ovarian Insufficiency
CRP: C-Reactive Protein
GnRH: Gonadotrophin-Releasing Hormone
LH: Luteinizing Hormone
FSH: Follicle-Stimulating Hormone
NK cells: Natural Killer Cells
DHEA: Dehydroepiandrosterone
CNS: Central Nervous System
ANS: Autonomic Nervous System
5-HT: Serotonin
ECS: Endocannabinoid System
ROS/RNS: Reactive Oxygen Species/ Reactive Nitrogen Species
RA: Rheumatoid Arthritis
